# Spatially Varying Associations of Neighborhood Disadvantage with Alcohol and Tobacco Retail Outlet Rates

**DOI:** 10.3390/ijerph19095244

**Published:** 2022-04-26

**Authors:** David C. Wheeler, Joseph Boyle, D. Jeremy Barsell, Trevin Glasgow, F. Joseph McClernon, Jason A. Oliver, Bernard F. Fuemmeler

**Affiliations:** 1 Department of Biostatistics, Virginia Commonwealth University, Richmond, VA 23298, USA; boylejr@vcu.edu; 2 Department of Health Behavior and Policy, Virginia Commonwealth University, Richmond, VA 23298, USA; barselldtj@vcu.edu (D.J.B.); trevin.glasgow@vcuhealth.org (T.G.); bernard.fuemmeler@vcuhealth.org (B.F.F.); 3 Department of Psychiatry and Behavioral Sciences, Duke University School of Medicine, Durham, NC 27705, USA; francis.mcclernon@duke.edu (F.J.M.); jason-oliver@ouhsc.edu (J.A.O.); 4 Stephenson Cancer Center, University of Oklahoma Health Sciences Center, Oklahoma City, OK 73104, USA; 5 Department of Psychiatry and Behavioral Sciences, Oklahoma State University Center for Health Sciences, Tulsa, OK 74107, USA; 6 Massey Cancer Center, Virginia Commonwealth University, Richmond, VA 23298, USA

**Keywords:** alcohol, tobacco, retail outlets, neighborhood disadvantage, spatially varying effects

## Abstract

More than 30% of cancer related deaths are related to tobacco or alcohol use. Controlling and restricting access to these cancer-causing products, especially in communities where there is a high prevalence of other cancer risk factors, has the potential to improve population health and reduce the risk of specific cancers associated with these substances in more vulnerable population subgroups. One policy-driven method of reducing access to these cancer-causing substances is to regulate where these products are sold through the placement and density of businesses selling tobacco and alcohol. Previous work has found significant positive associations between tobacco, alcohol, and tobacco and alcohol retail outlets (TRO, ARO, TARO) and a neighborhood disadvantage index (NDI) using Bayesian shared component index modeling, where NDI associations differed across outlet types and relative risks varied by population density (e.g., rural, suburban, urban). In this paper, we used a novel Bayesian index model with spatially varying effects to explore spatial nonstationarity in NDI effects for TROs, AROs, and TAROs across census tracts in North Carolina. The results revealed substantial variation in NDI effects that varied by outlet type. However, all outlet types had strong positive effects in one coastal area. The most important variables in the NDI were percent renters, Black racial segregation, and the percentage of homes built before 1940. Overall, more disadvantaged areas experienced a greater neighborhood burden of outlets selling one or both of alcohol and tobacco.

## 1. Introduction

More than 30% of cancer related deaths are related to tobacco or alcohol use [1]. The majority (55–66%) of cancer deaths in females that are attributable to alcohol are from breast cancer, while the majority (53–71%) of alcohol-attributed cancers in males are from the aero-digestive tract [2]. In addition, tobacco use has been linked to a variety of cancers, including cancers of the lung, urinary system, aero-digestive tract, and myeloid leukemia [3,4]. Reducing, controlling and/or restricting access to these cancer-causing products, especially in communities where there is a high prevalence of other cancer risk factors, has the potential to improve population health and reduce the risk of specific cancers associated with these substances in more vulnerable population subgroups.

One policy-driven method of reducing access to these cancer-causing substances is to monitor and regulate where and how these products are sold. Specifically, the placement and density of businesses selling tobacco and alcohol within neighborhoods could be reviewed and better regulated to address systemic issues (over representation within certain communities) that may contribute to an increase of sales of these products. States and local communities have the legal ability to influence the sale of tobacco and alcohol through zoning policies and granting licenses. Change at the state and community levels (e.g., reduce exposure and access to the products and any marketing, target social norms that promote use of the products) can possibly lead to individual-level changes in risky health behaviors (e.g., frequent tobacco and alcohol use) [5,6,7]. To inform such efforts, several studies have now assessed the relationship between socio-spatial demographics and retail density [8,9], and the strength of the association between retail density and increased consumption of cancer-causing products [10]. Consistently, studies have shown that both tobacco retail outlet (TRO) and alcohol retail outlet (ARO) density relative to the population are greater in neighborhoods having a lower median income and higher percent of minority residents [10,11,12]. The literature generally supports an association between greater density of these retail outlets and the greater use of tobacco and alcohol products among those who live in these neighborhoods [12,13].

Within this context of examining the associations between TROs and AROs and other variables, the associations have been examined separately in most studies, rather than examining the intersection of these types of product sales. This is a limitation because stores that sell both tobacco and alcohol, tobacco and alcohol retail outlets (TAROs), could have stronger associations with neighborhood deprivation than either of TROs or AROs independently. In other words, analyzing only TROs and AROs fails to estimate the neighborhood burden of TAROs.

In previous work, Wheeler et al. aimed to overcome this limitation to better understand the extent to which neighborhood socio-demographic characteristics relate to both the overlapping and distinct spatial distributions of TROs, AROs, and TAROs. They used a novel extension of the Bayesian index model that included a shared component for the spatial pattern common to all three types of outlet relative risks, outlet-specific spatial components for relative risks, and neighborhood disadvantage index (NDI) effects that varied by outlet type [14]. The Bayesian index model is a relatively new approach for estimating neighborhood disadvantage or deprivation indices [10,14,15,16,17,18] that considers the association with the outcome and can adjust for covariates and residual confounding through random effects. In Wheeler et al. [14], the NDI was estimated by a weighted combination of neighborhood socioeconomic status (SES) indicators (e.g., education, income, poverty) and racial/ethnic segregation (e.g., black population segregation). The results of that study included significant positive associations between TROs, AROs, TAROs and neighborhood disadvantage in block groups in North Carolina from the Bayesian shared component index model with associations that differed across outlet types. Regarding spatial patterns of outlets, the relative risks for each outlet type varied by levels of population density (e.g., rural, suburban, urban) and the shared component of relative risk had notable spatial heterogeneity.

While the study by Wheeler et al. found varying NDI effects by outlet type and population density and spatial structure in the relative risks for AROs, TROs, and TAROs, it did not consider that the spatial structure could be partially explained by neighborhood disadvantage effects that varied over space. In fact, there are no Bayesian index models with spatially varying coefficients in the literature, as previous specifications of Bayesian index models assume a constant regression effect for the index [10,14,15,16,17,18] over space. Regression models with spatially varying effects have been used previously in several fields including geography [19], ecology [20], hedonic price analysis [21], and epidemiology [22,23,24,25,26,27,28] to model nonstationarity in regression relationships. Nonstationarity reflects differences in a statistical relationship or confounding that varies across space or time [29]. Commonly used models for spatial nonstationarity are Bayesian spatially varying coefficient models and frequentist geographically weighted regression. One example of a rationale for using spatially varying coefficient models in hedonic price modeling is that the value of an additional bathroom means more in a highly dense market such as New York City compared with a rural market in Kansas where space is at less of a premium [19]. In a regression model of housing price, this means that the effect of the number of bathrooms would vary within a large market and over multiple markets. In an example in epidemiology, geographically weighted regression was used to identify spatially varying relationships between local urologist density and prostate cancer mortality at the county level in eastern and southern states in the US, where the strength of association varied regionally [22]. The region with the largest estimated decrease in prostate cancer mortality consisted of counties in the southern Mississippi River states of Arkansas, Louisiana, and Mississippi. In these counties, all else being equal, a one-unit increase in urologist density (one urologist per 100,000 men) was associated with a with lower prostate cancer mortality than in counties with coefficients closer to zero. These areas had higher prostate cancer mortality rates and relatively low urologist density. Therefore, these areas could be targeted for increasing the supply of urologists, as it associated with the largest predicted improvement in prostate cancer mortality.

Within the context of the present study, ignoring nonstationarity of the relationship between neighborhood disadvantage and outlet rates may be problematic because it assumes consistency of the relationship across space, and this may underestimate or inaccurately estimate the effect for neighborhood disadvantage in some areas. Therefore, in this study we propose using a new Bayesian spatially varying coefficient index model to model nonstationarity in neighborhood disadvantage effects related to TRO, ARO, and TARO rates over block groups in North Carolina. Specifically, we used a Bayesian index model with spatially varying effects to explore the hypothesis that NDI effects could vary over North Carolina to explain variation in outlet rates. We also sought to identify the geographic areas that were significantly elevated in NDI effects for each of the three outlet types to identify disparities in the neighborhood burden of tobacco and alcohol outlets.

## 2. Materials and Methods

### 2.1. Study Context 

We analyzed data from the state of North Carolina for our study. North Carolina does not require a license for retailers to sell tobacco products. However, the North Carolina Alcoholic Beverage Control (ABC) regulates the general sale of alcohol in the state. Retailers seek permits to sell beer and wine, but only state-run ABC stores are able to sell liquor. When considering whether to issue beer and wine permits to retailers, the ABC Commission considers several factors, such as the number of existing permits that have been granted in a neighborhood, parking availability, potential traffic problems, the types of existing businesses in the neighborhood, and the proximity of the applying retailer to schools and churches [30].

### 2.2. Study Data

#### 2.2.1. Tobacco and Alcohol Retail Outlets

We obtained data from two different sources to determine the TROs, AROs, and TAROs in North Carolina. The first data source we used was the National Establishment Time Series (NETS), which is a private, longitudinal, and geocoded record of all types of businesses in the United States. We utilized a previously developed algorithm to identify TROs [31] from this database. The algorithm filtered entries by their North American Industry Classification System codes to exclude businesses that were not TROs, deleted duplicate records, and removed records for TROs in years after state- or municipality-specific bans were enacted on the sale of tobacco products by certain business types. The second data source, for ARO data, was the North Carolina ABC Commission. The state’s ABC maintains a publicly-available database [30] of businesses that have received licenses to sell alcoholic beverages, including liquor stores that are run by the ABC. We took the business entries from this database that retained active and off-premise licenses to sell alcohol, in order to avoid including different types of businesses such as bars and restaurants. We geocoded AROs from their business address using the ArcMap software from ESRI and then standardized several variables in the data to maximize syntactical similarity between the TRO and ARO databases, such as converting all text to upper case, using a common street suffix, and excluding store identification numbers from business names. We then counted the number of TROs, AROs, and TAROs in each block group, a process which we have described in more detail previously [14]. At the end of this process, each block group had a count of businesses that were just TROs, just AROs, or TAROs. Across all the block groups in the state, we identified 7289 TROs, 4859 AROs, and 4631 TAROs. More details about the process for counting the number of outlets are also available elsewhere [14].

#### 2.2.2. Sociodemographic Data

We constructed neighborhood disadvantage indices using five-year estimates (2014–2018) of nine social and demographic variables collected at the census block group level by the American Community Survey (ACS), an annual survey representative of the US population that includes questions related to home ownership, income, and education, among other themes. We chose the time period 2014–2018 in order to overlap with the extent of the outlet data. The social and demographic variables included: Black population segregation, Hispanic population segregation, percent with income to poverty ratio < 1, percent households receiving public assistance income, percent renter occupied housing units, percent homes constructed in 1939 or earlier, percent with less than a high school degree, percent of households in poverty, and per capita income. The Black segregation variable was defined as the percent of Black population in a block group divided by the state average percent Black population. Hispanic segregation was defined similarly. We have used similar sociodemographic variables and segregation measures [18,32,33,34] to estimate neighborhood disadvantage indices previously [10,16]. We began with 6127 block groups in North Carolina and removed 15 that had no population and 29 others that had missing covariates to reach an analysis set of 6083. In this set of block groups, there were a total of 7279 TROs, 4849 AROs, and 4621 TAROs.

### 2.3. Statistical Analysis

We fit hierarchical Bayesian regression models to explain the observed variation in the rates of the outlet types (TRO, ARO, TARO), assuming that in the *i*th block group, the count of the *k*th outlet type was distributed as $y_{ik}∼Poisson\;\left(\theta_{ik}E_{ik}\right)$. For each block group, the modeled relative risk parameter was $\theta_{ik}$ and the expected count was $E_{ik}$, which we defined as the overall rate $r_{k}$ for the *k*th outlet type multiplied by the population in the block group $p_{i}$. The new Bayesian spatially varying coefficient index regression model of the log relative risk was:

(1) $$\log(\theta_{ik})=\alpha_{k}+\beta_{ik}\left(\sum_{j=1}^{C}w_{j}q_{ij}\right)$$

where $\alpha_{k}$ is the outlet-specific intercept and $\beta_{ik}$ is the outlet-specific, spatially-varying effect for the neighborhood disadvantage index in the *i*th block group. In each block group, the neighborhood disadvantage index was defined as a weighted combination $\overset{C}{\underset{j=1}{\sum }}w_{j}q_{j}$ of the deciles $q_{1},\;\ldots ,\;q_{c}$ of the sociodemographic variables $x_{1},\;\ldots ,\;x_{c}$, with the weights $w_{1},\;\ldots ,\;w_{C}$ being estimated in the model as part of the model parameters. The weight $w_{j}$ signifies the relative importance of the $j_{th}$ variable in the neighborhood disadvantage index. The index approach for estimating neighborhood disadvantage has been used previously [16,18,34]. We used deciles to account for different scaling of and correlations between the variables to mitigate the effect of outliers, and to acknowledge the uncertainty in ACS values. We used C=9 variables in the index. We defined the sociodemographic variables to reflect a hypothesized positive association of the index with relative risk (i.e., that greater disadvantage was associated with greater relative risk). We re-defined per capita income to have a positive association with rates based on univariate correlations with the outlet rates, inverting this variable with the formula $\max\left(x\right)-x_{j}$, where $x_{j}$ is the value of the variable.

The Bayesian model specification was completed with prior distributions for the parameters. The outlet-specific intercepts followed an improper uniform distribution $\alpha_{k}\sim dflat()$. The NDI regression coefficients were given a multivariate intrinsic conditional autoregressive (MVCAR) prior $\beta_{ik}\sim MVCAR(\Omega )$, which has been used previously to model spatially varying effects [35,36]. The precision matrix Ω for the MVCAR received a Wishart prior, Ω~Wishart(R,K), where R is a KxK diagonal matrix with 0.2 on the diagonal and K=3. The multivariate CAR prior allows the regression coefficients for one outlet type to be correlated over space (e.g., block groups) and for the regression coefficients to be correlated across outlet type. The inverse of Ω is the variance-covariance matrix for the regression coefficients, Σ. The index weights $w_{j}\;$ were given a Dirichlet prior with parameters $\alpha =\left(\alpha_{1},\ldots ,\alpha_{C}\right)$. The Dirichlet prior was selected because it assures that the index weights $w_{j}\in \left(0,1\right)$ and $\overset{C}{\underset{j=1}{\sum }}w_{j}=1$.

We estimated all model parameters using Markov chain Monte Carlo (MCMC) sampling, burning in 50,000 iterations and retaining an additional 10,000 iterations with a thinning parameter of one from two chains. We evaluated convergence in the MCMC chains for parameters of interest using the Gelman-Rubin diagnostic, considering a parameter to have converged if its Gelman-Rubin statistic was less than 1.2 [37]. We exponentiated the regression coefficients to get relative risk estimates for the neighborhood disadvantage index. The 95% credible interval was used to determine the statistical significance of the neighborhood disadvantage index relative risks, where there was a significant positive effect if the lower bound of the interval exceeded the value of 1. We also compared the distributions of the NDI variables and the disadvantage index between the block groups with and without significantly positive NDI effects using Welch’s *t*-test. To determine the amount of correlation between NDI relative risks among outlet types, we used the variance-covariance matrix components to calculate the conditional correlation between pairs of outlet types. We fit the Bayesian models using R2OpenBUGS [38] in the R computing environment [39].

## 3. Results

**Figure 1:**
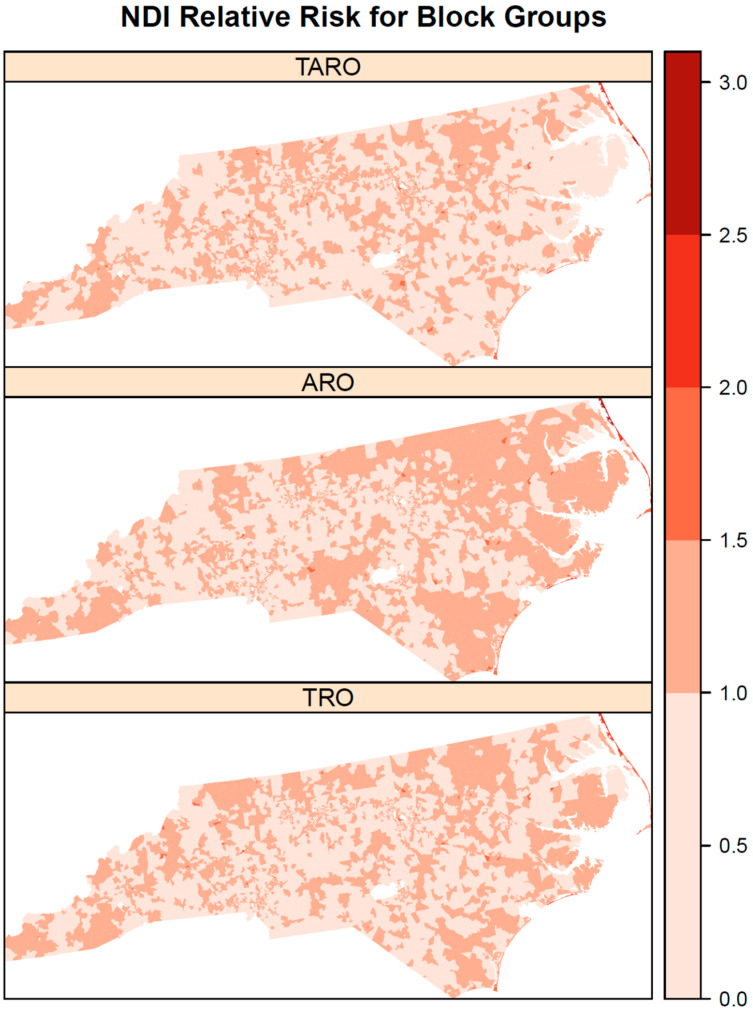
Posterior medians for neighborhood disadvantage index relative risks by outlet type for tobacco retail outlet (TRO), alcohol retail outlet (ARO), and tobacco and alcohol retail outlet (TARO). The legend is on the relative risk scale.

**Figure 2:**
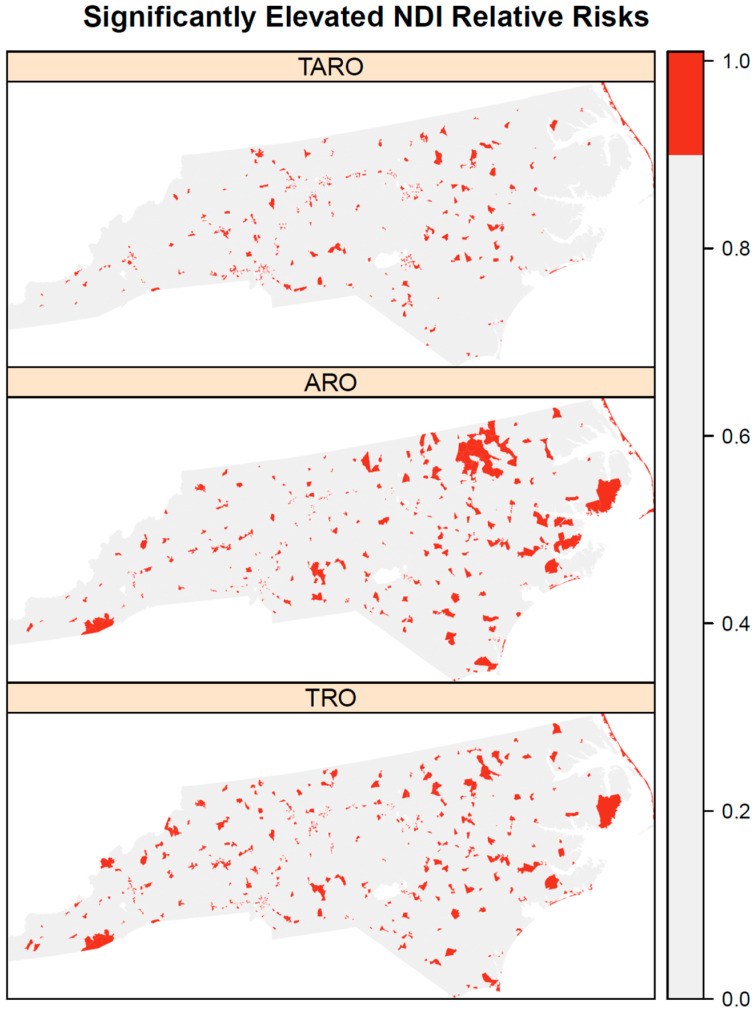
Areas with significantly elevated neighborhood disadvantage index effects by outlet type for tobacco retail outlet (TRO), alcohol retail outlet (ARO), and tobacco and alcohol retail outlet (TARO). The legend indicates red for 95% credible intervals that are above 1.

**Figure 3:**
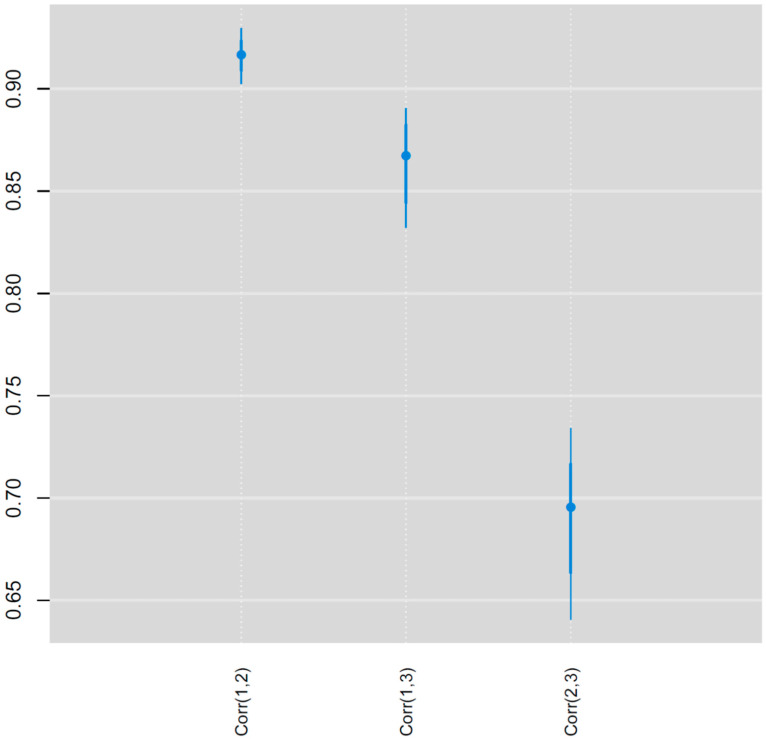
Posterior medians (circles) and 95% credible intervals for the conditional correlation in neighborhood disadvantage index regression coefficients by outlet type, where 1 = TROs, 2 = AROs, and 3 = TAROs.

The posterior median NDI relative risks show substantial variation over space for all three outlet types (Figure 1). Some correlation in the NDI relative risks between outlet types was evident, particularly with high relative risks in the northeastern coastal area (i.e., Outer Banks) for all three outlet types. In addition, all three outlet types also have areas of elevated NDI relative risk in southwestern North Carolina. However, there are varying amounts of correlation between NDI relative risks for pairs of outlet types (Figure 2). AROs and TROs were most highly correlated (posterior median = 0.92), followed by TROs and TAROs (posterior median = 0.87), and then AROs and TAROs (posterior median = 0.69). These correlations reinforce the observed visual patterns in Figure 1. For both TROs and AROs, there are areas of elevated NDI relative risk in northeastern North Carolina and the central and southern coastal areas. In contrast, TAROs have lowered NDI relative risks in northeastern North Carolina. The block groups with significantly elevated NDI relative risks according to 95% credible intervals (Figure 3) also show that the patterns are more similar for AROs and TROs than for either of these with TAROs. Both AROs and TROs have more block groups with significantly elevated NDI effects in the southwest and northeast of North Carolina. These store types show more clustering in elevated NDI effects than do TAROs.

**Figure 4:**
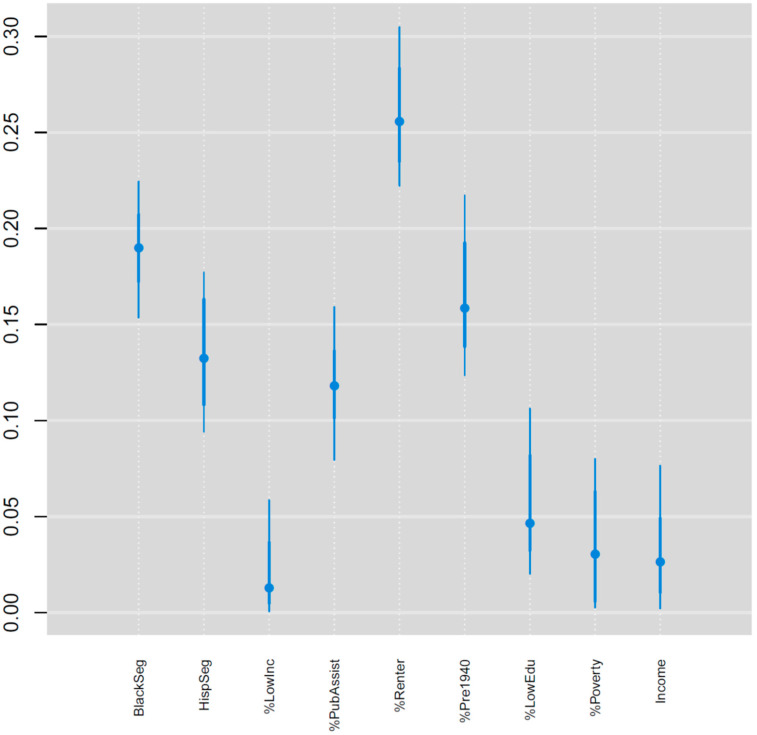
Posterior medians (circles) and 95% credible intervals for the neighborhood disadvantage index weights.

**Figure 5:**
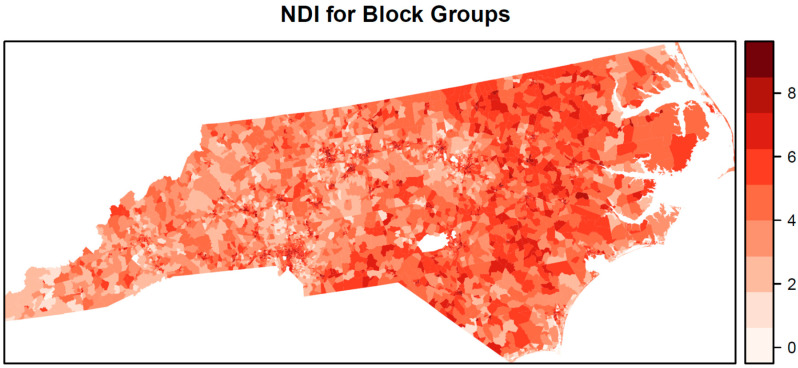
Neighborhood disadvantage index for block groups estimated from the Bayesian spatially varying coefficient index model.

For the estimated NDI, the weights for the nine components within the NDI (Figure 4) show that percent renter (0.26), black racial segregation (0.18), and percent of homes built before 1940 (0.16) were the three most important variables in the NDI and together accounted for 0.60 of the total weight. Hispanic segregation (0.14) and percent receiving public assistance (0.12) were the other components receiving more than a priori equal weight (0.11). The estimated NDI had a greater concentration of higher values in the eastern half of the state compared with the western half (Figure 5). However, neighborhood deprivation was also high in metropolitan areas located in the center and western half of the state.

To gain understanding about the areas with significantly elevated NDI relative risks, we compared the distributions of the NDI and deciles of the NDI variables between the set of block groups with significantly elevated NDI relative risks and those without. The Welch *t*-test results showed significant differences (*p* < 0.01 for all tests) in the means of the index and all nine of the NDI variables for TROs, where the index and NDI variables were all higher in the set with significantly elevated NDI relative risks (see Supplemental Material Table S1). For AROs, test results were similar, with all tests significant (*p* < 0.05) except for Hispanic segregation, which was slightly higher in the set without elevated NDI relative risks. For TAROs, the tests were all significant (*p* < 0.01) with the NDI and NDI variable means being higher in the set with significantly elevated NDI relative risks. When looking at the distributions of the NDI and the deciles of the NDI variables, there was a shift to high values in the set with significantly elevated NDI relative risks compared with the set without significantly elevated NDI relative risks. Thus, areas with significantly elevated NDI relative risks were generally more disadvantaged areas. Therefore, while the NDI effect is not equal over space, it is generally larger in the more deprived areas. However, there were some block groups with significantly elevated NDI relative risk (≥1.8) and low values (<5) of the NDI (see Supplemental Material Figure S1).

## 4. Discussion

In this paper, we aimed to estimate spatially varying relationships between a neighborhood disadvantage index and TRO, ARO, and TARO rates using a novel Bayesian spatially varying coefficient index model to help address nonstationarity of the relationship between neighborhood disadvantage and tobacco and retail outlets. Our results showed substantial variation in the associations between the estimated neighborhood disadvantage index and the rates of each outlet type, with some notable similarities and differences in effects by outlet type. All three outlet types (AROs, TROs and TAROs) had significantly elevated NDI relative risks in the northeastern shore area of the Outer Banks. However, TROs and AROs had more significantly elevated NDI effect areas in the northeast and southeastern coast compared with TAROs, and AROs in particular had a notable cluster of significantly elevated effects in northeastern North Carolina near the state line. Overall, the NDI effects for TROs and AROs were more strongly correlated with each other than with the effects for TAROs. The block groups with significantly elevated NDI relative risks had larger values of the NDI index and NDI variables (e.g., percent renters) overall than did the block groups without significantly elevated NDI relative risks, indicating that more disadvantaged areas experienced a greater neighborhood burden of outlets selling one or both of alcohol and tobacco overall. The most important variables in the NDI were percent renters (i.e., low home ownership), Black racial segregation, and percentage of homes built before 1940 (i.e., old housing stock).

Our study considered the spatially varying associations of neighborhood deprivation with TROs, AROs, and TAROs simultaneously. Prior research has centered on only TROs [12,40] or only AROs [7,41], with few to none examining their simultaneous combination (TAROs). Research on TROs and AROs is not limited to the US. For example, in North India [42], both TROs and AROs were geocoded by physically walking in neighborhoods with a map app determining the coordinates of the outlets. While the prevalence of smoking and drinking were associated with TRO density, the at-risk health behaviors were not associated with ARO density. In Scotland, another study [8] examined both TROs and AROs and their associations with neighborhood deprivation. The result from that study that density of the retail outlets is associated with neighborhoods experiencing greater deprivation is consistent with our results. However, in that study, neighborhood deprivation was assessed only by income and by the percentage receiving government benefits and assistance. Furthermore, no previous studies have considered these associations to vary over space. By allowing the associations of NDI and the three outlet types to vary over space, we found that more disadvantaged areas overall experienced a greater neighborhood burden of outlets selling one or both of alcohol and tobacco, which highlights a disparity that is important for public health.

In this study, we employed novel analytic methods to assess the spatially varying relationships of alcohol and tobacco outlets with neighborhood sociodemographic characteristics. We extended the Bayesian index model to have spatially varying regression coefficients for multiple outcome rates. The Bayesian index regression model provides more informative output than the traditional dimension reduction techniques used in the literature [43,44], such as principal component analysis (PCA) and factor analysis. The interpretation of the components produced by PCA and factor analysis are not easy to interpret and do not account for the association with the outcome. In contrast, our Bayesian index model estimates the index weights considering associations with the outcomes and produces easily interpretable weights indicating relative importance, some of which can be effectively zero for unimportant variables. In our case, only five variables showed up as being relatively important while four had small weights. We have demonstrated previously that employing an index approach to at once estimate the NDI as well as its health effect leads to significantly improved model goodness-of-fit than constructing the index with principal components analysis [33]. Another strength of our study is its extension of the Bayesian index regression model to accommodate several related outcomes at once, as we allowed spatially varying NDI effects to be correlated across store types. We used the information in the three outlet types to estimate the neighborhood disadvantage index for all block groups together, but allowed the effect of the NDI to uniquely vary spatially for each outlet type, which in this case was useful as illustrated by the differences in patterns of effects (Figure 1) and significantly elevated effects for outlets by type (Figure 2). In our previous work, we did not allow the NDI effects to vary over space for each outlet type and therefore could not uncover our current finding that more disadvantaged areas experienced a greater neighborhood burden of outlets selling tobacco and/or alcohol.

One of the benefits of the Bayesian index model approach is that it can empirically determine the most important components in the index for explaining the outcome variable. Indeed, in our study not all variables in the index were equally important in explaining the association between neighborhood disadvantage and outlet rates (e.g., Figure 4). Select variables reflecting neighborhood socioeconomic disadvantage, such as low home ownership and older housing stock, were more important than income and poverty related variables. Racial and ethnic segregation also received more weight than the income and poverty variables. Our finding of an association with percent renters may be related to cigarette product marketing and use among youth and young adults, as youth are more likely to rent their homes [45] and be exposed to and use cigarette products [46]. Our results align to some extent with those reported from other geospatial studies conducted in New Jersey [47], Boston, Massachusetts [48], and a recent US based study [49] where race and ethnicity were important variables for TRO density. However, household income was less important in our analysis than in the New Jersey study, where percent Hispanic population was the dominant demographic factor associated with TRO density, followed by median household income and percentage of Black population. One possible explanation for differences in variable importance across studies is that our study considered outlets selling alcohol and tobacco and alcohol together in addition to just tobacco outlets. Furthermore, the relative importance of these variables in explaining retail outlet density may reflect different local and state tobacco control policies, as well as differences in the statistical methods used. More progressive locales may better implement policies that affect retail outlet density through excise taxes and prohibitory regulations, such as restrictions on placement within certain distances of specific locations. Therefore, the continued characterization of retail outlet placement is necessary for understanding how neighborhood characteristics are associated with TRO, ARO, and TARO density over time and across geographic areas.

A discussion of the limitations of the study is also warranted. First, we operationalized the concept of neighborhood disadvantage in a block group through the use of one fixed set of sociodemographic variables at that block group. We did not consider other covariates in the block group, or variables from a larger spatial unit containing the block group, such as the census tract or county. Secondly, we emphasize that our findings are limited to the state of North Carolina. Future research should adopt the methods of our study to other geographic regions. Different relationships may hold in different contexts, such as policies regulating sales and zoning of these products, as well as of the social norms regarding alcohol and tobacco. Finally, we drew TRO and ARO business data from large registries, and the tobacco data source required an additional algorithm to classify stores as likely selling tobacco. Though we performed extensive checks to verify the accuracy of these listings and of our resulting analysis datasets, some error that is attributable to the large business databases (unregistered businesses, or those not captured by the database) is unavoidable.

## 5. Conclusions

In summary, our use of Bayesian spatially varying coefficient index models uncovered significant positive associations between neighborhood disadvantage and rates of tobacco, alcohol, and tobacco and alcohol outlets in North Carolina and identified clear geographic disparities in the neighborhood burden of these outlets. The factors driving the significant associations include low levels of home ownership, black racial segregation, and old housing stock. The findings in our study help to shed light on the dominant neighborhood factors that are associated with the heterogeneous distribution of tobacco and alcohol retail outlets, which are themselves environmental factors that drive smoke exposure and alcohol use. A deep understanding of both the individual- and environmental-level determinants of smoke exposure is a priority for public health, especially given the increased potential for adverse health outcomes in newborns and children [50]. Future studies should center on economic and educational factors in disadvantaged areas, as well as their connection to smoking and alcohol use, and more broadly on the structural inequalities that encourage the use of these products in these areas. The modeling approach presented in this paper should be useful in other geographic areas for uncovering disparities in neighborhood burden of tobacco and alcohol retail outlets and the factors most related to the disparities. Furthermore, they help support and justify policies and approaches that limit retail outlets in certain neighborhoods in order to reduce the apparent systemic injustice of placement of high rates of tobacco or alcohol retail outlets among minoritized communities.

## Data Availability

Publicly available datasets were analyzed in this study. NETS data are available from Don Wells at dwalls2@earthlink.net. ARO data are available at https://abc.nc.gov/Permit/Retail (accessed on 1 March 2021). ACS data are available at https://www.census.gov/programs-surveys/acs (accessed on 1 March 2021).

## Supplementary Materials

The following supporting information can be downloaded at: https://www.mdpi.com/article/10.3390/ijerph19095244/s1, Figure S1: Block groups with significantly elevated neighborhood disadvantage index (NDI) relative risk >= 1.8 and NDI < 5; Table S1: Two-sample *t*-test results for deciles of neighborhood disadvantage index (NDI) variables between block groups with significantly elevated NDI effects and without significantly elevated NDI effects.
