# Supplementary material for: Spatially Varying Associations of Neighborhood Disadvantage with Alcohol and Tobacco Retail Outlet Rates
Source: Int J Environ Res Public Health. 2022 Apr 26;19(9):5244. doi: 10.3390/ijerph19095244 (PMC9101141; doi:10.3390/ijerph19095244)

Supplemental Material

Spatially Varying Associations of Neighborhood Disadvantage with Alcohol and Tobacco Retail Outlet Rates

David C. Wheeler[1], Joseph Boyle[1], D. Jeremy Barsell[2], Trevin Glasgow[2], F. Joseph McClernon[3], Jason A. Oliver[3,4,5], Bernard F. Fuemmeler[2,6]

[1]Department of Biostatistics, Virginia Commonwealth University, Richmond, VA 23298

[2]Department of Health Behavior and Policy, Virginia Commonwealth University, Richmond, VA 23298

[3]Department of Psychiatry and Behavioral Sciences, Duke University School of Medicine, Durham NC 27705

[4]Stephenson Cancer Center, University of Oklahoma Health Sciences Center, Oklahoma City, OK 73104

[5]Department of Psychiatry and Behavioral Sciences, Oklahoma State University Center for Health Sciences, Tulsa, OK 74107

[6]Massey Cancer Center, Virginia Commonwealth University, Richmond, VA 23298

Corresponding author: David C. Wheeler, email: dcwheeler@vcu.edu, phone: 804-828-9827, address: One Capitol Square, 830 East Main Street, Richmond, VA 23298-0032

Table S1. Two-sample t-test results for deciles of neighborhood disadvantage index (NDI) variables between block groups with significantly elevated NDI effects and without significantly elevated NDI effects.

	TRO			ARO			TARO		
**Variable**	**Significantly Elevated**	**Not Significantly Elevated**	**P-value**	**Significantly Elevated**	**Not Significantly Elevated**	**P-value**	**Significantly Elevated**	**Not Significantly Elevated**	**P-value**
Black segregation	5.017	4.432	0.000	5.291	4.419	0.000	5.492	4.395	0.000
Hispanic segregation	4.854	4.453	0.001	4.445	4.506	0.647	5.146	4.431	0.000
Income to poverty ratio < 1	5.455	4.374	0.000	5.456	4.402	0.000	5.535	4.390	0.000
Public assistance	4.839	4.455	0.001	4.785	4.471	0.015	4.895	4.458	0.001
Renter	6.129	4.286	0.000	5.881	4.359	0.000	6.503	4.288	0.000
Pre 1940 housing	5.311	4.393	0.000	5.316	4.417	0.000	5.096	4.437	0.000
Less than high school degree	5.272	4.398	0.000	5.273	4.421	0.000	5.360	4.409	0.000
Poverty	5.485	4.370	0.000	5.564	4.391	0.000	5.578	4.386	0.000
Per capita income (reversed)	5.136	4.416	0.000	5.213	4.427	0.000	5.362	4.409	0.000

Figure S1. Block groups with significantly elevated neighborhood disadvantage index (NDI) relative risk >= 1.8 and NDI < 5

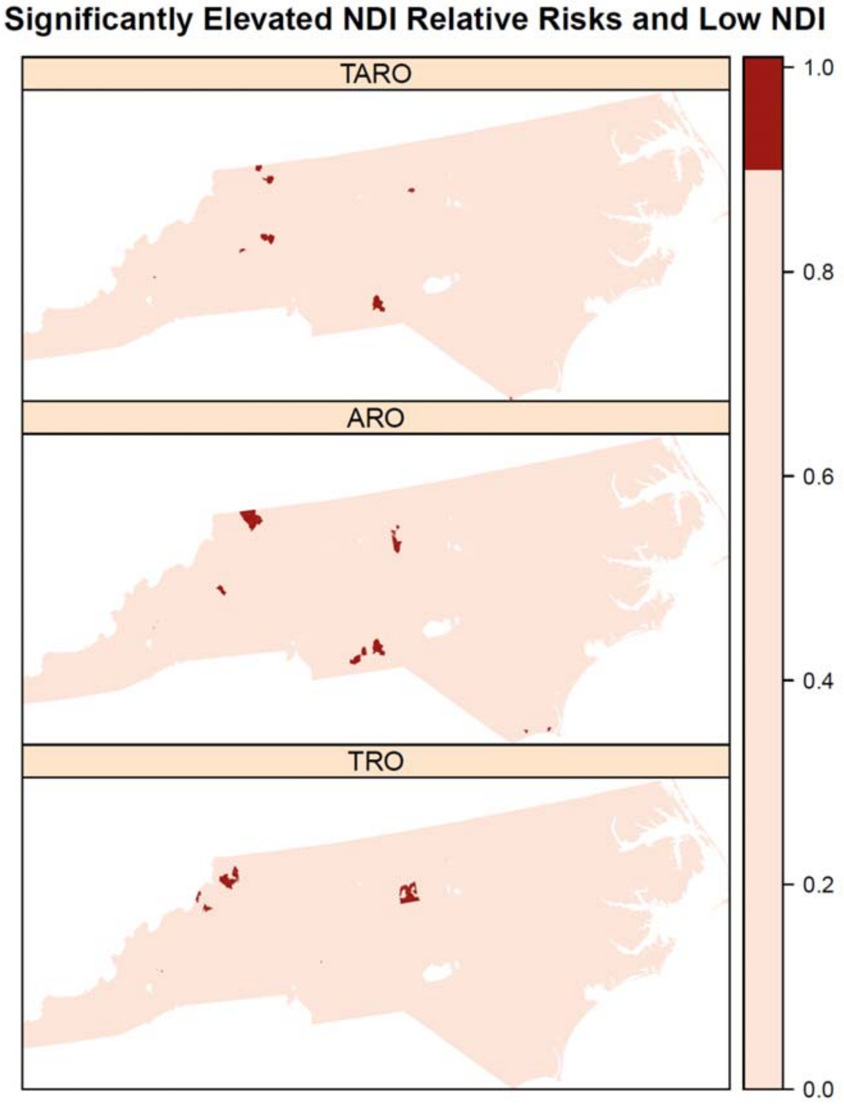

Supplement: Supplementary file 1 [file ijerph-19-05244-s001.zip › ijerph-1663817-supplementary.pdf]
